# Wearable-Based Assessment for Relapse Prediction Following Repetitive Transcranial Magnetic Stimulation for Depression: Protocol for a Feasibility Study (WARN-D Study)

**DOI:** 10.2196/89466

**Published:** 2026-09-21

**Authors:** Clarissa Dine, Zakariya Rekkas, Benicio N Frey, Sridhar Krishnan, Dante Duarte, Fabiano A Gomes, Michael S B Mak, Caroline Wanderley Espinola

**Affiliations:** 1 Department of Psychology, Neuroscience and Behaviour, McMaster University, Hamilton, ON, Canada; 2 Faculty of Health Sciences, McMaster University, Hamilton, ON, Canada; 3 Department of Psychiatry & Behavioural Neurosciences, McMaster University, Hamilton, ON, Canada; 4 Mood Disorders Treatment and Research Clinic, St. Joseph’s Healthcare Hamilton, 100 West 5th St, Hamilton, ON, L9C 1G2, Canada, 1 (905) 522-1155; 5 Signal Analysis Research Lab, Toronto Metropolitan University, Toronto, ON, Canada; 6 Seniors Mental Health Program, St. Joseph’s Healthcare Hamilton, Hamilton, ON, Canada; 7 Department of Psychiatry, University of Toronto, Toronto, ON, Canada; 8 Centre for Addiction and Mental Health, Toronto, ON, Canada

**Keywords:** transcranial magnetic stimulation, digital mental health, depression, feasibility study, wearables

## Abstract

**Background:**

A significant proportion of patients with major depressive disorder do not achieve remission after 2 antidepressant trials and are considered to have treatment-resistant depression (TRD). Repetitive transcranial magnetic stimulation (rTMS) is an effective treatment for TRD. However, relapse rates among remitters within the first year post treatment are significant, and there are no validated markers of relapse. Wearable devices have shown positive results for longitudinal monitoring of health metrics, and may be a promising tool for early detection of relapse following rTMS treatment.

**Objective:**

This study aims to evaluate the feasibility of a wearable device (Oura Ring) to monitor individuals receiving rTMS treatment for depression. We will also explore the utility of wearable-derived data as preliminary markers of treatment response and depressive relapse in a 6-month follow-up period.

**Methods:**

This single-arm pilot study will recruit 25 outpatients with a major depressive episode receiving rTMS at 2 tertiary hospitals in Ontario, Canada. Participants will be required to use a smart ring throughout the treatment course and during the 6-month follow-up. Clinical assessments including the Montgomery-Åsberg Depression Rating Scale (MADRS), Patient Health Questionnaire-9 (PHQ-9), Generalized Anxiety Disorder-7 (GAD-7), Insomnia Severity Index (ISI), Posttraumatic Stress Disorder Checklist for *DSM-5* (PCL-5), and World Health Organization-Five Well-Being Index (WHO-5) will be collected at baseline, treatment end, and 3- and 6-month follow-ups, alongside biweekly PHQ-9 and GAD-7 scores. The primary outcomes will be defined by feasibility measures (ie, recruitment, adherence, retention, missing data, and usability). Exploratory outcomes will include the assessment of preliminary associations between wearable-derived features and clinical outcomes and depressive relapse.

**Results:**

The study was funded in December 2025, and data collection will commence following research ethics approval. At the time of manuscript submission, the study protocol was under review at the Research Ethics Board of the participating institutions, and no study-related activities, including participant recruitment, had started. Data analysis and dissemination of findings will occur following completion of data collection.

**Conclusions:**

This study will provide initial evidence on the feasibility and utility of wearable-based digital phenotyping in individuals receiving rTMS for TRD. Our findings will inform the design of future large-scale studies aimed at wearable-supported relapse prevention and precision monitoring in depression care.

## Introduction

### Background

Major depressive disorder (MDD) affects more than 280 million individuals worldwide and is a leading contributor to global disability, with a lifetime prevalence estimated at 15%‐20% [1]. Beyond its prevalence, MDD places a heavy burden on individuals and health care systems, contributing to impaired quality of life, high treatment costs, and an increased risk of suicide [2]. Roughly one-third of patients do not obtain response after 2 or more antidepressant trials, a condition referred to as treatment-resistant depression (TRD) [3]. Compared with non-TRD depression, these patients face greater illness burden, more frequent relapses, and a markedly higher risk of suicidal behavior [3,4].

Repetitive transcranial magnetic stimulation (rTMS) is a well-tolerated, noninvasive brain stimulation technique that delivers magnetic pulses to the cortex, inducing changes in cortical excitability and promoting synaptic plasticity [5], which may eventually lead to an improvement in depressive symptoms. rTMS is considered a first-line treatment for TRD [2], with response and remission rates of 41%‐56% [2,6] and 26%‐28% [6,7], respectively. Despite its superior effectiveness compared with antidepressants, a significant number of patients successfully treated with rTMS ultimately relapse within the first year following treatment [8,9]. The duration of TMS antidepressant effects is variable, and sustained remission tends to decrease over time [9,10]. Approximately one-third of patients experience recurrence of depressive symptoms in the first 3 months post treatment [9,11], with relapse rates increasing to 50% within 6‐12 months [9]. However, a naturalistic study reported a substantially higher relapse rate of 77% at 6 months following an acute rTMS course [11]. These discrepancies in relapse rates may partially reflect differences in study populations, as “real-world” cohorts often include patients with greater clinical complexity, including higher levels of treatment resistance and psychiatric comorbidity, compared with clinical trials [4]. Differences in definition of relapse, clinical outcome measures, and use of maintenance treatment protocols across studies may also contribute to the heterogeneity in reported relapse rates [9]. Fortunately, most patients who experience relapse tend to respond to a new treatment course of rTMS [10,12,13], with some reports indicating successful retreatment in more than 80% of cases [14] and clinical benefits being reported across different rTMS treatment protocols [12,15].

Although some clinical variables were identified as potential predictors of response (eg, number of treatment failures and baseline severity) [16,17] and relapse (eg, comorbid anxiety and residual symptoms) [18] to rTMS, the evidence to support their use is limited, and there are currently no established biomarkers of rTMS treatment outcomes [10,19]. Emerging evidence suggests that cardiovascular and autonomic markers may have potential in this context. A recent meta-analysis demonstrated that rTMS influences cardiovascular markers by reducing resting heart rate (RHR) and increasing heart rate variability (HRV) [20]. Similarly, one pilot study reported that increased HRV correlated with improvement in depressive symptoms following 15 Hz rTMS [21], while another found that lower RHR during treatment was associated with treatment response in accelerated low-frequency (LF) rTMS, although this association did not remain significant after correction for multiple comparisons [22]. Despite these promising findings, prior studies have largely relied on isolated assessments of physiological parameters rather than continuous or longitudinal monitoring across rTMS treatment and follow-up periods. Moreover, none of these studies evaluated autonomic and cardiovascular markers as predictors of relapse following rTMS treatment. Taken together, these findings support the potential utility of physiological markers while also highlighting an important unmet need for scalable and low-burden strategies capable of identifying patients at a higher risk of relapse. Early identification and longitudinal monitoring of these individuals could allow for the detection of subtle changes in physiological signals and other parameters preceding depressive relapse, allowing timely treatment reinitiation before further symptom deterioration and potentially improving functionality and quality of life.

Digital phenotyping refers to the “moment-by-moment quantification of the individual-level human phenotype in-situ using data from smartphones and other personal digital devices” [23,24]. Early digital phenotyping studies, especially those reliant on frequent active input, have often faced challenges such as a high proportion of missing data and engagement barriers, frequently due to participant forgetfulness or perceived burden [25]. A recent remote study in a large sample of frontline health care workers found the daily burden of wearable sensor usage was estimated at only 2‐4 minutes per day, supporting the feasibility of low-burden, passive monitoring approaches [26]. While passive wearable data completion rates remain high, active survey completion rates tend to decrease across the duration of long-term digital phenotyping studies [25,27]. Furthermore, different smartphone-based systems that rely on active monitoring or sensitive contextual passive data may have variable adherence and data completeness [28]. For instance, iOS and Android smartphones differ significantly in sampling rate consistency; iOS devices generally have more regular sensor sampling rates than Android, which means data resolution can vary depending on the device used [29]. These limitations emphasize the value of passive, continuous “always-on” sensing technologies [30].

Wearable devices, such as smart rings, address this gap by continuously collecting high-resolution data on activity, physiological, and behavioral patterns with minimal user input required [25,31]. For example, smart wearables integrate multiple sensors to collect data on physical activity, sleep patterns, and physiological signals including heart rate (HR), HRV, and body temperature [32]. When combined with active self-reported assessments, these passive data create multimodal near-real-time datasets that can capture a person’s lived experience with higher ecological validity and temporal resolution than standard clinical assessments [33]. These dense datasets hold promise for the application of machine learning (ML) models to predict treatment outcomes [34]. In our study, we have decided to use a smart wearable named Oura Ring due to its successful use in previous longitudinal studies, with demonstrated feasibility and high accuracy for physiological data collection. This wearable has been extensively adopted in health monitoring and mental health research with high acceptability [25,26,30,35-39], high adherence (80%‐92%), and low attrition rates being reported even in long-term longitudinal studies [26,36]. The feasibility of this wearable for data collection in mental health has been established in previous studies in individuals with depression, anxiety, and bipolar disorder [40-42]. Furthermore, the Oura Ring has demonstrated superior accuracy for RHR and HRV measurement compared with other commercial wrist-worn devices, including WHOOP and Polar [38,43]. Specifically, Oura devices show the highest agreement with electrocardiogram (ECG) for nocturnal RHR and HRV (Lin’s concordance correlation coefficient [CCC] up to 0.99) among all devices studied [38]. Similarly, in comparison with polysomnography (PSG), the Oura Ring outperformed commercial wrist wearables (Fitbit Sense 2 and Apple Watch Series 8) in classifying sleep stage, exhibiting substantial epoch-by-epoch agreement (Cohen κ >0.61) and showing no significant differences from PSG for total duration estimates of light, deep, or rapid eye movement (REM) sleep [38].

Despite the high accuracy of wearable data, the feasibility of wearable-based monitoring during rTMS treatment and follow-up has not yet been investigated. This is a significant gap considering the high relapse rates seen in this population and the need for low-burden longitudinal monitoring during follow-up. Although digital biomarkers have been examined as predictors of depressive relapse in other depressed samples [44,45], to our knowledge, only one study has explored the prediction of rTMS treatment response using smartphone data [46], and none has explored the use of wearables for relapse prediction in this specific population. This proof-of-concept study collected active data (symptom surveys and cognitive tasks), passive data (number of steps and relative physical positioning), and metadata (phone usage and screen time) using a smartphone app on a sample of 19 individuals receiving rTMS. The authors reported promising results for response prediction with an area under the curve (AUC) of 0.911 for the predictive model using combined passive and active data collected throughout the treatment course [46]. However, the study did not incorporate sleep metrics, high-resolution physical activity data, or physiological parameters, nor did it evaluate the utility of digital phenotyping for relapse prediction in this patient population. Another pilot study investigated the use of actigraphy measures to assess the effects of rTMS treatment on sleep (total sleep time, sleep efficiency, and wake after sleep onset [WASO]), and activity parameters (number of steps per day and energy expenditure) of 14 patients with MDD [47]. The authors reported significant improvement in depressive symptoms and a significant increase in the number of steps per day with no difference for sleep parameters or energy expenditure. However, this study only assessed actigraphy data from 7 days before rTMS treatment until the end of the rTMS course, and follow-up data collection was not performed to monitor symptom relapse or potential delayed effects [48] of rTMS. Furthermore, it did not collect physiological data, including RHR and HRV, both of which have been associated with response to rTMS in previous studies [20-22]. Together, these findings highlight the potential of digital interventions to monitor mental health and predict treatment outcomes in depression, while underscoring the need for studies exploring the use of wearables, which are generally associated with higher acceptability and accuracy with lower burden than smartphone apps [49,50].

### Objectives

The overarching aim of this pilot study is to investigate the feasibility of a wearable device (Oura Ring) to monitor individuals receiving rTMS treatment for depression. Our primary objective is to assess recruitment rates, usability, adherence, retention rates, and the proportion of missing data of the study wearable device. We will additionally explore the utility of wearable-derived measures collected during the 6-month follow-up period as preliminary predictors of antidepressant response and remission as well as biomarkers of relapse among rTMS responders. Furthermore, we will examine potential associations between sleep parameters, trauma-related symptoms, and clinical outcomes (ie, response and remission) as well as depressive relapse.

## Methods

### Study Design

**Figure 1.**
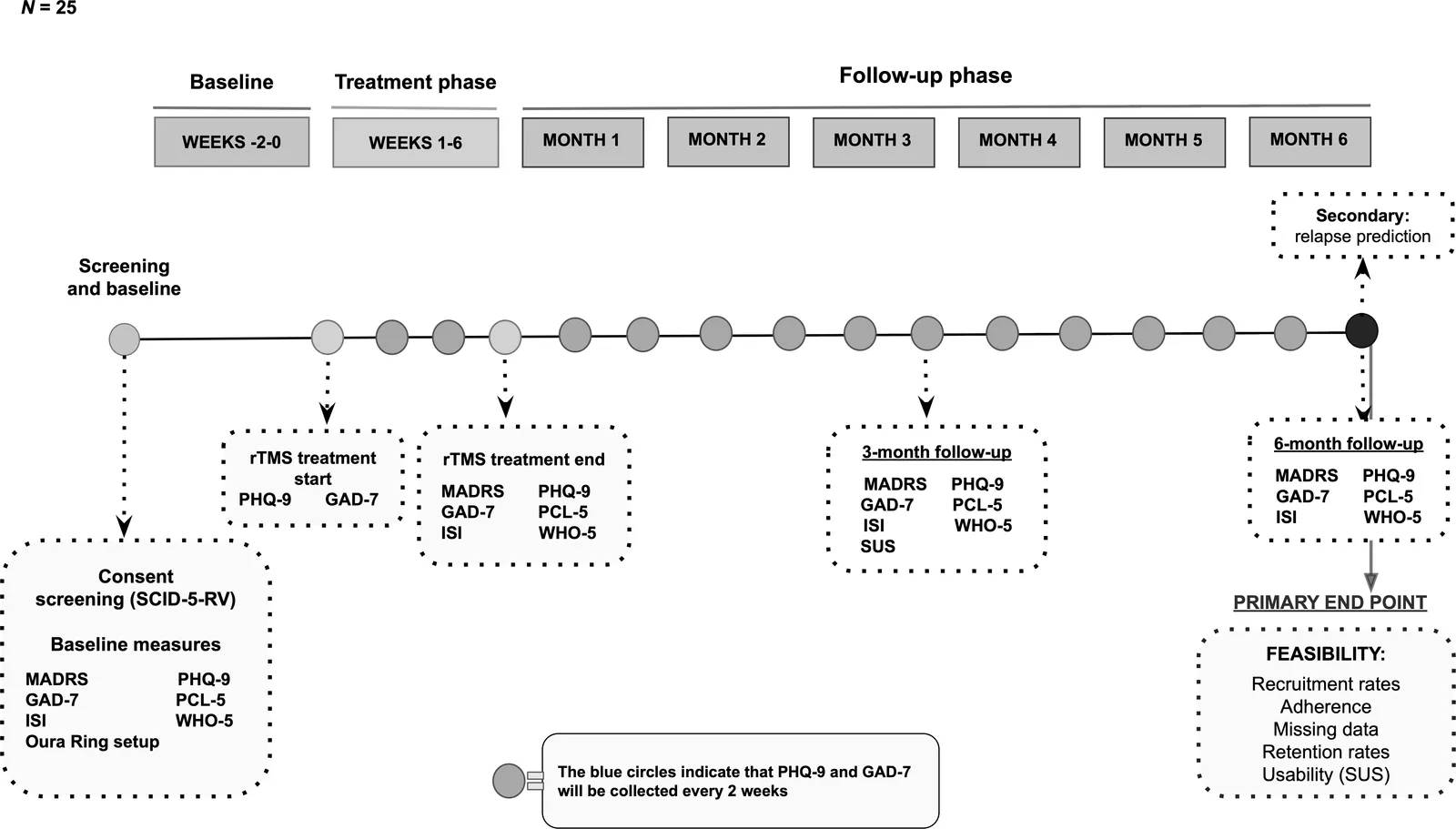
Study design with clinical assessments. GAD-7: Generalized Anxiety Disorder-7; ISI: Insomnia Severity Index; MADRS: Montgomery-Åsberg Depression Rating Scale; PCL-5: Posttraumatic Stress Disorder Checklist for *DSM-5*; PHQ-9: Patient Health Questionnaire-9; rTMS: repetitive transcranial magnetic stimulation; SCID-5-RV: Structured Clinical Interview for *DSM-5 Disorders*-Research Version; SUS: System Usability Scale; and WHO-5: World Health Organization-Five Well-Being Index.

This is a 2-site prospective observational pilot open-label study to assess the feasibility of a wearable device to continuously collect passive health data from individuals receiving rTMS for a major depressive episode (MDE). The study will have a duration of 24 months, with each participant being enrolled in the study for an approximate period of 8 months. Participants will be required to use the study wearable throughout their participation in the study—from baseline, during the rTMS treatment course until the end of the 6-month follow-up period. Approval from the local Research Ethics Board (REB) of the participating institutions will be obtained before the commencement of any study-related activities. Details on the study design and assessments to be conducted are provided in Figure 1 and Multimedia Appendix 1, respectively. The study investigators did not receive any kind of support from the wearable device manufacturer.

### Participants and Settings

**Figure 2.**
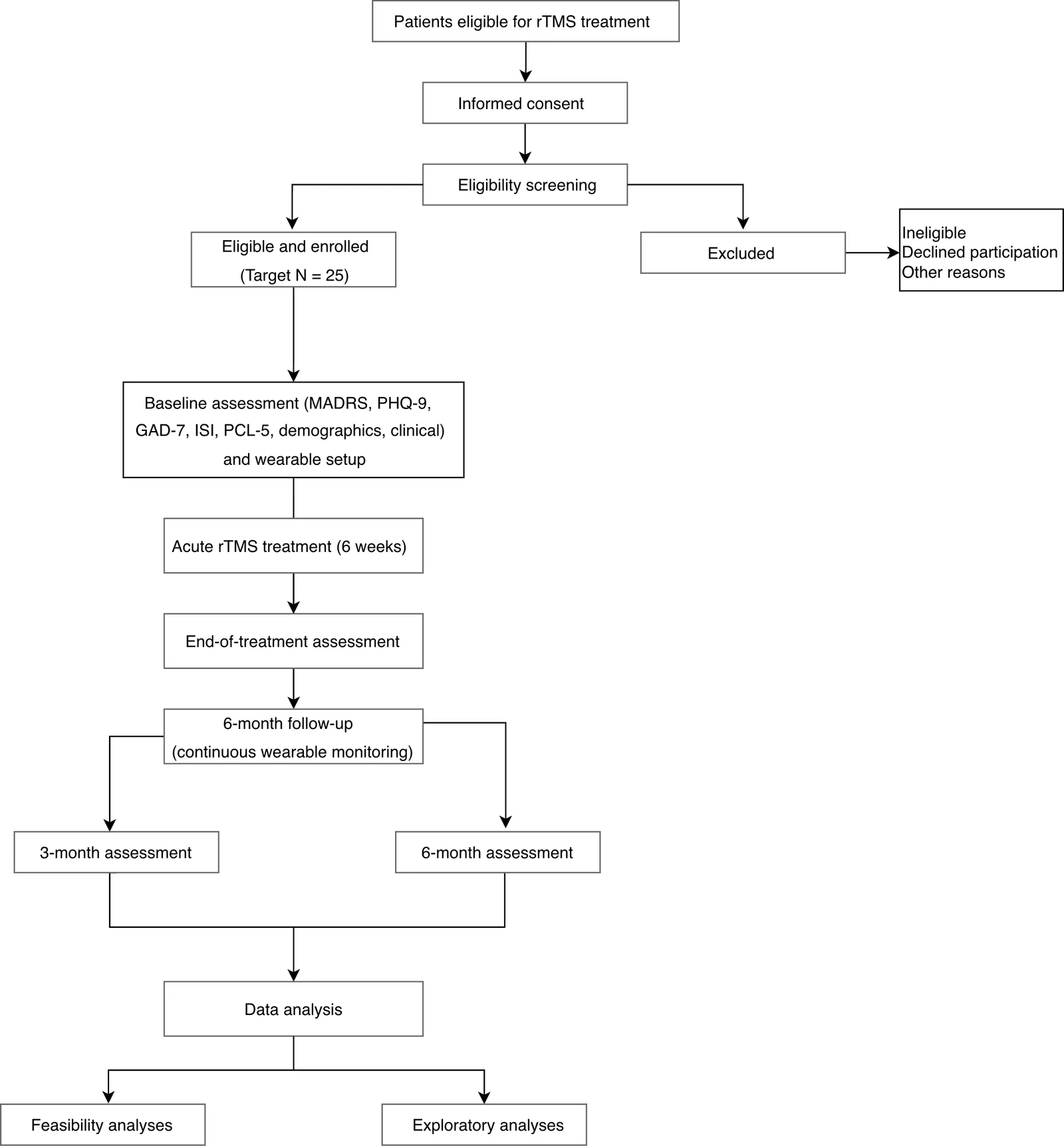
Study flow diagram.

This study will be conducted at St. Joseph’s Healthcare Hamilton (SJHH) and St. Joseph’s Healthcare London (SJHL), 2 tertiary academic hospitals specialized in mood disorders in Ontario, Canada. Over a 12-month period, 25 eligible participants referred to rTMS will be recruited primarily from the TMS Clinics at both institutions. The inclusion criteria are: (1) outpatients aged 18 years or older eligible to receive treatment with rTMS, (2) a diagnosis of an MDE confirmed by the Structured Clinical Interview for *DSM-5* Disorders-Research Version (SCID-5-RV), (3) a score of >20 in the Montgomery-Åsberg Depression Rating Scale (MADRS), (4) ability to provide written consent, (5) willingness and ability to complete self-reported assessments including sufficient fluency in English, and (6) willingness to use the study wearable device for the duration of the study. Exclusion criteria include (1) presence of a comorbid psychiatric condition considered to be causing more impairment than the depressive disorder, (2) presence of psychotic symptoms, (3) active suicidal ideation with plan and/or intent, (4) comorbid moderate or severe alcohol or substance use disorder in the last 3 months, (5) presence of a medical or neurological condition that in the opinion of the investigator poses a risk to participating in the study, (6) allergy to any component of the study wearable, and (7) presence of a sensory impairment that may interfere with the ability to participate in the study. Potential participants will provide informed consent prior to their enrollment in the study using a REB-approved Informed Consent Form (ICF). The study flow diagram is illustrated in Figure 2.

### Feasibility Outcome Definitions

Feasibility will be demonstrated if the following a priori criteria are met: (1) a recruitment rate of at least 50% of eligible individuals approached for participation, (2) adherence of at least 80%, defined as participants providing valid wearable data for ≥80% of the expected monitoring period, (3) a retention rate of at least 80%, defined as the proportion of enrolled participants who complete the study through the 6-month follow-up, (4) missing data of 25% or less for wearable data and questionnaire-based responses, and (5) acceptable usability of the study wearable, defined as a mean System Usability Scale (SUS) score of at least 68.

### Clinical Outcome Definitions

In this study, we have adopted well-established definitions of clinical outcomes. Response is defined as a reduction of at least 50% from the MADRS score at any follow-up assessment. Remission is defined as a MADRS score of <10 at the end of treatment or during follow-up. Relapse is defined as either a MADRS total score of >22 at any study visit following remission, or a Patient Health Questionnaire-9 (PHQ-9) score of >10 on 2 consecutive assessments separated by at least 2 weeks.

### Baseline and Follow-Up Assessments

At the screening and baseline visit, we will collect demographic (eg, age, sex, gender identity, ethnicity, and education) and clinical data (diagnosis, psychiatric comorbidities, medical history, antidepressant treatment history, and concomitant medications). Study assessments will include the following (Multimedia Appendix 1):

1. Depressive symptoms: MADRS [51] and PHQ-9 [52].
2. Anxiety symptoms: Generalized Anxiety Disorder-7 (GAD-7) [53].
3. Sleep: Insomnia Severity Index (ISI) [54].
4. Trauma-related symptoms: The Posttraumatic Stress Disorder Checklist for *DSM-5* (PCL-5) [55].
5. Well-being: World Health Organization-Five Well-Being Index (WHO-5) [56].
6. Wearable usability: SUS [57].

Beyond depressive symptoms, we will examine the presence of anxiety and trauma-related symptoms to explore their association with wearable features and clinical outcomes. Characterization of comorbid symptoms will provide a more comprehensive understanding of the study sample, given the high prevalence of these symptoms among individuals with MDD [58-60], particularly those with TRD [61,62]. Additionally, the presence of comorbid anxiety and trauma-related symptoms may be associated with distinct behavioral and physiological phenotypes [63-67] from individuals without comorbidities.

Participants will undergo baseline screening and clinical assessments prior to initiating rTMS treatment, followed by continuous wearable monitoring throughout both the treatment and follow-up phase. Clinical and self-reported assessments will be collected at predefined intervals across treatment and follow-up visits. MADRS assessments will occur at baseline, treatment end, 3-month follow-up, and at study end point (ie, 6-month follow-up). PHQ-9 and GAD-7 questionnaires will additionally be collected biweekly from baseline through the end of the 6-month follow-up period using the REDCap platform (Vanderbilt University). REDCap is a secure web app for managing and storing surveys in research studies [68]. We will also collect ISI, PCL-5, and WHO-5 assessments at baseline, treatment end, 3-month follow-up, and study end point. Wearable usability will be assessed by the SUS at the 3-month and 6-month follow-up assessments. During the screening and baseline visit, participants will be requested to start using the wearable device, which should be worn continuously throughout their participation in the study (ie, from baseline until the end of the 6-month follow-up) on the nondominant hand. The study wearable will collect passive physiological, sleep, and activity data continuously throughout the study period to support longitudinal monitoring and exploratory analyses of relapse-related digital phenotyping patterns. Concomitant medications will be recorded at baseline, updated at the end of acute rTMS treatment, and at the 3- and 6-month follow-up assessments. At each assessment, participants will be asked whether any psychotropic medications have been initiated, discontinued, or modified since the previous study visit. Medication name, dose, frequency, and the approximate date of any change will be documented when available. Multimedia Appendix 2 provides a schedule of the study visits.

### rTMS Treatments

Participants will receive rTMS treatment as part of the standard of care. The primary protocol used at both TMS clinics is the US Food and Drug Administration (FDA)–approved intermittent theta burst stimulation (iTBS) protocol, which consists of 50 Hz triplet bursts repeated at 5 Hz at an intensity of 120% of the resting motor threshold (RMT) over the left dorsolateral prefrontal cortex (DLPFC), with a stimulus train duration (on cycle) of 2 seconds and an intertrain interval (ITI, off cycle) of 8 seconds, for a total of 600 pulses delivered over 3 minutes and 9 seconds [7]. In a few cases, the standard FDA-approved high-frequency (HF) 10 Hz rTMS may be used instead for individuals with poor tolerability to iTBS or who have a history of previous good response to HF-rTMS. This protocol consists of 10 Hz with a train duration of 4 seconds, with a 26-second ITI delivered on the left DLPFC at 120% RMT stimulation intensity over 37.5 minutes for a total of 3000 pulses per session [69]. For both iTBS and HF protocols, a minimum stimulation intensity of 100% RMT will be accepted based on individual tolerability. Treatments at SJHH will be delivered with a Magstim Horizon Performance (Magstim Company Ltd), while in SJHL a MagVenture MagPro X100 (MagVenture A/S) will be used. A 70-mm figure-of-eight coil will be used at both treatment sites, with an EZ-cooled coil at SJHH and a Cool-B70 at SJHL.

### Wearable Device Measures

Participants will be provided with a commercially available wearable device named Oura Ring (Oura Health Ltd) that continuously collects physiological, sleep, and activity data. Data will be collected longitudinally with minimal burden to the participant and without obstruction of one’s daily life. A detailed description of the metrics collected by the study wearable is provided in Multimedia Appendix 3.

### Data Collection, Security, and Confidentiality

A designated member of the study team will create email accounts that will be used to set up anonymous user accounts on the wearable device mobile app, thereby ensuring deidentified data capture. After the participant has provided consent, they will be given their unique credentials to log in to the Oura app (Oura Health Oy).

A master linking log will store the names and the corresponding study credentials of research participants. Only this log will contain their personally identifiable information, and this will be kept in a secure, password-protected device separate from the rest of the study data.

Demographic information and data from assessment scales will be collected and stored on REDCap using a password-protected account. Wearable devices will be set up by a research member with a corresponding unidentified ID, and the research team will access the wearable manufacturer’s specific API to extract the wearable data from its respective servers using a CSV or JSON file. No personally identifiable data will be collected by the wearable devices, as each user will have a unique and deidentified email assigned to them. Demographic data (eg, age, gender, occupation, education, medications, psychiatric history, and so on) collected as part of the screening and baseline visit will be collected and stored separately from the data from the wearable account. Demographic information will only be linked to the wearable data during data analysis. Therefore, participant data will be collected anonymously on the wearable app, and deidentified data will be stored on its servers.

Data collected from the wearable devices will be transmitted to and stored on secure, proprietary servers maintained by the device manufacturer. Data ownership will not follow a single-owner model. Participants will remain the data subject and will control whether their data are shared. Once a participant signs the ICF where they consent to share data with our institutions, our research team will become the controller of this study data and its downstream use in compliance with applicable law. The wearable company may act as the processor or service provider for the hosting, storage, and technical administration of the wearable data environment, as stated in the ICF. At the end of the study, we will request all wearable data to be deleted from the manufacturer’s servers. Deidentified study data will be stored in a secure, password-protected institutional server for 10 years, following the local institution’s policy and in accordance with regulatory ethical requirements.

Wearable data will be transmitted from the participant’s phone to the manufacturer’s secure cloud. Oura operates its primary infrastructure on Amazon Web Services (AWS) with encryption in transit (TLS 1.2+) and at rest (AES-256). This study will comply with applicable institutional privacy policies and Ontario’s Personal Health Information Protection Act (PHIPA), which governs the collection, use, and disclosure of personal health information in the province where this study will be conducted. The proposed use of the wearable platform, including data storage and any international transfer of data, is currently under review by the participating institutions’ REBs and will be implemented only following REB approval. The wearable manufacturer is a global company with servers located worldwide, so personal deidentified data may be processed on servers outside the participant’s country under approved international data-transfer frameworks. Additionally, the manufacturer ensures that participant data are protected with industry-leading privacy and security practices. Details on data collection, storage, and management are provided in the Oura Health Privacy Policy [70]. For compliance safeguards, the wearable platform is protected by Systems and Organization Controls (SOC) 2 Type II attestation and HITRUST security and compliance framework e1 certification, with encryption in transit and at rest and role-based access controls around participant data.

Our contingency plan for data recovery in case the manufacturer’s API is discontinued will consist of scheduled periodic exports (ie, biweekly) of study data from their servers to our institution repository, with the institution retaining those exported files as the study record rather than treating the commercial platform as the sole copy.

### Adverse Event Reporting and Risks

Participants may feel slightly burdened by the need to use the wearable device daily, to regularly open the wearable app to sync the data with its servers and/or to charge the device. A fully charged Oura Ring has a battery life of 5‐8 days [71]. Participants might also find the data summaries and insights provided by the wearable app to be upsetting or distressing, although previous studies have shown that wearables tend to be generally accepted in individuals with depression [40,72]. Additionally, participants may experience an allergic reaction to the materials in the wearable device. All adverse events will be documented and reported in accordance with institutional REB requirements.

As with all research studies that store confidential information, there is a risk that privacy may be breached. However, participant identifiable information will be locally stored in a password-protected shared drive in a folder that can only be accessed by the site investigator and the study research coordinator to mitigate privacy risks.

### Statistical Analysis

Descriptive statistics (eg, means, SDs, and proportions) will be used to summarize primary continuous outcomes, such as recruitment and retention rates, proportion of missing data, and adherence. For exploratory outcomes of relapse and response prediction, we will use linear mixed-effects models (LMMs) to account for repeated measures and interindividual variability over time. Sleep data will be analyzed using appropriate correlation techniques. We will also assess the quality of the recorded signals to understand how noise characteristics shift across different daily activity and motion conditions. Time domain, frequency domain, and joint time and frequency domain features will be extracted to support this characterization and evaluate signal quality under varied use conditions. Changes to medications in use over the course of this study will be summarized descriptively and may be incorporated as covariates in exploratory analyses where feasible, while recognizing the limited sample size and feasibility nature of the study. A clear and standardized artifact management approach, including motion artifact suppression and adaptive filtering, will also be applied to ensure reliable performance during ambulatory monitoring and longitudinal use [73].

Additionally, exploratory ML techniques (eg, pattern classification and anomaly detection techniques) will be applied to investigate preliminary patterns and trajectories of PHQ-9, MADRS, GAD-7, and PCL-5 scores using wearable data. To reduce dimensionality and mitigate the risk of overfitting, regularized regression approaches such as Least Absolute Shrinkage and Selection Operator (LASSO) will be explored for feature selection. Following dimensionality reduction, exploratory ML methods including random forest, support vector machines (SVMs), and gradient boosting methods (eg, XGBoost [Extreme Gradient Boosting]) will be evaluated where supported by data structure and availability. Building on previous work with ensemble classifiers, feature selection, and XGBoost [74,75], model performance will be assessed using resampling approaches, such as leave-one-out cross-validation (LOOCV) or repeated k-fold cross-validation, acknowledging that these metrics may be unstable in small samples and subject to optimism bias. Therefore, results will be interpreted descriptively and will not be considered evidence of predictive validity or model generalizability.

Exploratory performance metrics will include AUC, sensitivity, specificity, precision, *F*_1_-score, and calibration measures where feasible. Again, these metrics will be interpreted descriptively and cautiously and will not be considered evidence of predictive validity, generalizability, or clinical utility. Biweekly PHQ-9 and GAD-7 scores will be integrated as active digital phenotyping of longitudinal clinical variables within exploratory analyses. Specifically, we will explore lagged values, change scores, symptom trajectories, and symptom-worsening thresholds aligned with passive sensing windows. Missing data will be characterized by source, time point, and participant characteristics, and addressed using multiple imputation for questionnaire and covariate data, with complete-case sensitivity analyses. For wearable time-series data, we will use segmentation-based validity windows and appropriate imputation methods, including interpolation, window-level aggregation, and model-based approaches [76,77]. Given the pilot nature of the study and the reduced anticipated number of relapse events, predictive results from ML models will be exploratory and hypothesis-generating rather than intended to draw definite conclusions on clinical relevance.

Although the participant-level sample size is not designed for the validation of predictive models, this is appropriate for the primary feasibility objectives of this study, including recruitment, retention, adherence, data completeness, and usability. Nevertheless, wearable data collection will generate a large volume of repeated passive observations across the study period, including daily summaries, hourly summaries, and high-resolution physiological and activity data sampled at 5-minute intervals (Multimedia Appendix 3). This longitudinal structure results in thousands of repeated observations and is consistent with methodological approaches frequently adopted in digital phenotyping research. Importantly, our analyses are exploratory rather than aimed at the development or validation of clinically generalizable predictive models. These analyses aim to explore candidate digital markers of relapse or clinical response to be further investigated in a future adequately powered study. Performance results, despite using cross-validation techniques, will remain exploratory and should be interpreted with caution given the small sample size, the low anticipated number of relapse events, and the risk of overfitting. Where feasible, and contingent on sample size and privacy considerations, we will conduct exploratory disaggregated analyses by age group and gender to identify subgroup-specific trends.

### Sample Size Justification

To our knowledge, only a limited number of studies piloted digital phenotyping in rTMS-treated patients, including an actigraphy study with a sample size of 14 participants [47], as well as another pilot study using smartphone-based monitoring in a sample size of 19 participants [46]. Based on prior literature [46,47,78,79], we estimate that a sample of 25 participants will be sufficient for the primary feasibility aims of this pilot study. Additionally, using a digital phenotyping sample size calculator [80] and assuming a conservative attrition rate of 20%, a type I error of 0.05, a follow-up duration of 180 days, and a modest effect size (β₁=0.03), a sample size of 25 participants should provide a statistical power of approximately 85%. For our study, we adopted a conservative approach of 80% power.

## Results

The study was funded in December 2025. At the time of its submission, this study protocol was under review by the REB of both participating institutions (SJHH and SJHL). Participant recruitment and data collection will begin following ethics approval. Data analysis will occur after participants have completed the follow-up period.

## Discussion

### Principal Findings

Depression remains one of the leading contributors to global disability, and approximately one-third of patients develop TRD. Existing clinical predictors, such as comorbid anxiety or residual symptoms, have poor predictive value, and there are currently no validated biomarkers of relapse following rTMS treatment. These limitations underscore the need to identify objective, low-burden, and scalable approaches for early relapse detection. To our knowledge, this study will be the first to evaluate the feasibility of using a wearable device to passively collect relevant data for mental health monitoring in patients receiving rTMS. Wearables can collect physiological, activity, and behavioral metrics, including sleep time, HR, and HRV, WASO, and mobility features, which have been shown to be related to the presence of and changes in depressive symptoms [40]. Digital phenotyping may also help capture subtle day-to-day changes and fluctuations in passive behavioral and physiological features, which may provide insights into changes in mood and emerging signs of relapse beyond what can be detected by less frequent clinical assessments [81]. Advanced ML techniques may be useful for exploring complex relationships among behavioral, physiological, and activity data collected through wearable device sensors. In this context, ML methods may facilitate the identification of candidate digital markers associated with treatment outcomes and inform the future development of personalized relapse prediction models. Such approaches could complement conventional clinical assessments by leveraging longitudinal, naturalistic data collected outside of clinical settings [82]. Within the framework of precision psychiatry, passive digital monitoring may support more personalized approaches to care by enabling scalable, real-world symptom tracking and informing individualized intervention strategies. Smart rings and other unobtrusive wearables are particularly well suited for this purpose, as they are generally feasible, minimally burdensome, and compatible with routine, longitudinal data collection in naturalistic settings [83].

We anticipate that this study will demonstrate the feasibility of using the Oura Ring for continuous passive monitoring in individuals receiving rTMS for TRD, as reflected in adequate recruitment rates (> 50%), reaching a sample of 25 participants over 12 months across 2 sites; high wearable adherence (>80% based on prior research using Oura Ring); acceptable retention (>80%) across a 6-month follow-up period; low proportion (<25%) of missing wearable data and questionnaire responses; and favorable usability scores (ie, > 68) on the SUS. As an exploratory analysis, we aim to identify preliminary wearable-derived physiological (eg, HR, HRV, and body temperature) and behavioral (eg, number of steps) features associated with clinical outcomes, generating candidate digital biomarkers for validation in future adequately powered studies.

### Comparison to Prior Work

The anticipated feasibility of wearable monitoring in this TRD population is supported by a growing body of prior research using the Oura Ring. Previous longitudinal studies using this wearable have demonstrated high adherence and low attrition, supporting its acceptability for continuous passive data collection [26]. Moshe et al [40] successfully used the Oura Ring and smartphone data to predict symptoms of depression and anxiety in real-world settings, concluding that passive sensing can track symptom fluctuations with minimal user burden. Similarly, a larger-scale study by Mason et al [42] found that elevated distal body temperature measured via Oura Ring was significantly associated with depressive symptoms, illustrating the sensitivity of continuous physiological monitoring to predict mood states. Furthermore, the study by Matcham et al [44] extended this work to MDD and demonstrated that wearable-derived sleep features are associated with relapse risk. Collectively, these findings across similar samples including individuals with depressive and anxiety symptoms provide a strong basis for the anticipated feasibility of wearable-based monitoring in the TRD population in this study. We will expand this knowledge by examining the feasibility of this wearable device in individuals receiving rTMS and will additionally explore the identification of candidate digital biomarkers of treatment outcomes both across the rTMS treatment course and throughout a 6-month follow-up period.

Wearable-based features show promise as objective markers of changes in depressive symptoms. A recent meta-analysis found that rTMS is associated with an increase in HRV and a reduction in RHR, suggesting that autonomic nervous system function may represent a physiological correlate of treatment response [20]. Digital phenotyping offers the ability to capture subtle intraindividual fluctuations that may not be detected by less frequent clinical assessments. As such, it may enable the detection of early behavioral and physiological shifts that may precede clinical improvement or full syndrome relapse by days or weeks. Despite this potential, no previous study has longitudinally monitored individuals following an rTMS course using wearable technology, and none has investigated the utility of wearable-derived features for early relapse detection. This study aims to address these gaps by collecting continuous, high-resolution wearable data during the acute rTMS course and throughout a 6-month follow-up period, alongside biweekly self-reported questionnaires and regular clinician-rated assessments. Furthermore, the integration of passive wearable data with active survey data will create a multimodal dataset suitable for time-series and ML analyses, including anomaly detection techniques. This may facilitate the exploration of digital signatures associated with treatment outcomes and depressive relapse, while allowing a comparison of the predictive performance of passive and combined digital phenotyping methods.

### Strengths and Limitations

A major strength of this study is the integration of wearable-derived circadian and physiological features within a real-world rTMS treatment setting, enabling continuous high-resolution data collection alongside routine psychiatric care [31,84]. Feasibility outcomes, including adherence, retention, and usability, will provide important information regarding the acceptability of long-term wearable monitoring in individuals with TRD, the design of future multisite studies as well as the refinement and validation of analytical approaches for processing longitudinal digital phenotyping data [85]. The passive nature of wearable data collection may also overcome limitations commonly associated with smartphone-based ecological momentary assessments, including participant burden, declining engagement, and survey fatigue, potentially making wearable technologies a more scalable and sustainable approach for longitudinal symptom monitoring and prediction modeling in depression [81]. To our knowledge, this is the first study to evaluate the feasibility of integrating wearable-based digital phenotyping into rTMS treatment for TRD. Furthermore, the combination of continuous wearable data with clinical and self-reported assessments will generate a multimodal dataset that can be used to develop and explore preliminary prediction models of treatment response and relapse, while describing clinically relevant intraindividual behavioral and physiological variability associated with these outcomes [86]. Although exploratory, these analyses may help identify candidate digital biomarkers of treatment response and depressive relapse that can be refined and validated in larger prospective studies.

This study has several limitations. Given its small sample size, this study may not be powered enough to detect small yet significant associations between passive and active data. Additionally, this small sample size does not allow for building generalizable predictive models of treatment outcomes. As such, ML analyses in this study aim to identify preliminary digital signals that may inform future, adequately powered, predictive modeling studies. ML models tend to overfit in small datasets, especially in the context of multidimensional data [87,88]. As such, this study does not aim to generate robust predictive models; instead, it will investigate preliminary features to be explored in future studies. Another limitation is the risk of missing data due to nonadherence or technical failures, including nonwear periods or device syncing issues, respectively. Although previously published studies report overall high adherence to wearable monitoring, high rates of missing data—should it occur in this study—can introduce several forms of bias and reduce the reliability of predictive models [40]. Specifically, selection bias may occur if individuals who experience symptom worsening are less likely to consistently wear the device, leading to systematic underrepresentation of severe cases in the sample. Attrition bias may result if participants with greater disease burden or difficulty accessing treatment are more likely to discontinue device use, skewing the remaining sample toward milder or more adherent cases. Furthermore, missing data from nonwear periods or technical failures may selectively occur during specific times of day or activities, introducing measurement bias that distorts the true patterns of activity, sleep, and physiological parameters. Furthermore, although the accuracy of the Oura Ring has been validated for HR and sleep data collection [30,89,90], wearable-based data will likely differ from gold-standard techniques such as PSG and ECG. Moreover, although concurrent medication use will be collected at different time points and included in the analysis model, changes to medications during the study period may be underreported or occur between time points, which could be a potential confounder, particularly when assessing for depressive relapse during the follow-up period. Although participants are advised not to make changes to their medications during their rTMS course, given the observational nature of this study, medication changes are still allowed and may confound response to rTMS. Finally, participant recruitment from 2 specialized tertiary centers may limit the generalizability of our findings to broader populations, including community-based and primary care settings [83].

### Future Directions

This study will enable the development of larger multisite studies aimed at validating wearable-based digital biomarkers for predicting treatment outcomes and depressive relapse following rTMS treatment in more diverse patient populations and settings [91]. Early relapse detection may yield multiple clinical and research benefits. Real-time monitoring using wearable features could facilitate timely intervention and retreatment before symptom worsening, potentially reducing clinical deterioration and preventing hospitalizations and associated health care costs, while supporting functional recovery and quality of life [4,92]. Early detection of symptom worsening also has the potential to reduce suicide risk, which remains elevated in individuals with TRD [93]. Additionally, continuous wearable data collection also offers the opportunity to characterize response and relapse trajectories through advanced analytical methods including anomaly detection and ML techniques [73,82,94,95], thereby improving our understanding of changes in behavioral and physiological parameters that precede both clinical improvement and deterioration.

The findings from this pilot study will provide important feasibility metrics and preliminary effect size estimates to inform the design and sample size calculation of future, large-scale studies. Future applications may incorporate multimodal data such as smartphone-based ecological momentary assessments. Additionally, future studies should investigate the utility of individualized wearable monitoring in guiding booster or maintenance rTMS schedules to facilitate timely, targeted relapse-prevention interventions. Algorithms must also incorporate ethical and equity considerations, transparency, and be co-designed with stakeholders to support successful real-world implementation [82]. As digital phenotyping continues to evolve, prioritizing privacy, generalizability, and interdisciplinary collaboration will be essential to maximize the clinical applicability of ML-based predictive models in clinical psychiatry, including early identification of treatment responders and relapse prevention in MDD.

### Dissemination Plan

The findings of this study will be disseminated through peer-reviewed publications, conference presentations, and knowledge translation activities targeting researchers, clinicians, and individuals with living experience of depression. Results related to feasibility and preliminary exploratory associations between wearable-derived measures and clinical outcomes will be submitted to relevant national and international conferences in psychiatry, brain stimulation, biomedical engineering, and digital mental health. Study findings will be submitted for publication in peer-reviewed journals regardless of the direction or significance of the results. In addition, a summary of the study findings will be made available to participants upon request.

### Conclusion

This pilot study will be the first to examine the feasibility of integrating wearable-based digital phenotyping into the longitudinal monitoring of individuals receiving rTMS for TRD. It also has the potential to identify preliminary behavioral and physiological patterns associated with treatment response and depressive relapse. Despite the effectiveness of rTMS for depression, the high relapse rates following successful treatment underscore the need for more precise monitoring and understanding of patterns underlying relapse. By evaluating the feasibility, acceptability, and utility of continuous monitoring using the Oura Ring, this research will generate the foundational evidence to inform the design of future adequately powered studies and the development of wearable-derived digital biomarkers. Such biomarkers may ultimately support earlier identification of treatment response, detection of relapse risk, and more personalized follow-up care. If validated in larger studies, wearable-based monitoring could enhance clinical decision-making and facilitate timely interventions, with the potential to improve outcomes and reduce illness burden for individuals receiving rTMS for treatment-resistant depression.

## Supplementary material

10.2196/89466 Multimedia Appendix 1 Summary of baseline demographic, self-reported questionnaires, and clinical assessments.

10.2196/89466 Multimedia Appendix 2 Schedule of study visits.

10.2196/89466 Multimedia Appendix 3 Description of the metrics collected by the study wearable (Oura Ring).

## Abbreviations

AUC: area under the curve
AWS: Amazon Web Services
CCC: concordance correlation coefficient
DLPFC: dorsolateral prefrontal cortex
ECG: electrocardiogram
FDA: US Food and Drug Administration
GAD-7: Generalized Anxiety Disorder-7
HF: high frequency
HR: heart rate
HRV: heart rate variability
ICF: Informed Consent Form
ISI: Insomnia Severity Index
iTBS: intermittent theta burst stimulation
ITI: intertrain interval
LASSO: Least Absolute Shrinkage and Selection Operator
LF: low frequency
LMM: linear mixed-effects model
LOOCV: leave-one-out cross-validation
MADRS: Montgomery-Åsberg Depression Rating Scale
MDD: major depressive disorder
MDE: major depressive episode
ML: machine learning
PCL-5: Posttraumatic Stress Disorder Checklist for *DSM-5*
PHIPA: Personal Health Information Protection Act
PHQ-9: Patient Health Questionnaire, 9-item
PSG: polysomnography
REB: Research Ethics Board
REM: rapid eye movement
RHR: resting heart rate
RMT: resting motor threshold
rTMS: repetitive transcranial magnetic stimulation
SCID-5-RV: Structured Clinical Interview for *DSM-5* Disorders-Research Version
SJHH: St. Joseph’s Healthcare Hamilton
SJHL: St. Joseph’s Healthcare London
SOC: Systems and Organization Controls
SUS: System Usability Scale
SVM: support vector machine
TRD: treatment-resistant depression
WASO: wake after sleep onset
WHO-5: World Health Organization-Five Well-Being Index
XGBoost: Extreme Gradient Boosting
