# Supplementary material for: Wearable-Based Assessment for Relapse Prediction Following Repetitive Transcranial Magnetic Stimulation for Depression: Protocol for a Feasibility Study (WARN-D Study)
Source: JMIR Res Protoc. 2026 Sep 21;15:e89466. doi: 10.2196/89466 (PMC13592458; doi:10.2196/89466)
Supplement: Supplementary file 1 [file resprot-v15-e89466-s001.docx]

Multimedia Appendix 1. Summary of Baseline Demographic, Self-Reported Questionnaires and Clinical Assessments

| Category | Variables Collected | Frequency of Data Capture |
| --- | --- | --- |
| Demographics | Age, sex, gender, ethnicity, education, marital status, employment status, job classification, household information | Baseline |
| Clinical History | Primary Diagnosis (i.e., unipolar or bipolar depression)  Psychiatric Comorbidities  Previous Antidepressant Trials  Medical, Psychiatric, and Substance Use History | Baseline |
| Concomitant Medications | Medications in use | Baseline  Treatment End  3-Month Follow-Up  6-Month Follow-Up (Study End) |
| Assessment Scales | | |
| Depressive Symptoms (Clinician-Administered) | Montgomery-Åsberg Depression Rating Scale (MADRS) | Baseline  Treatment End  3-Month Follow-Up  6-Month Follow-Up (Study End) |
| Depressive Symptoms (Self-Report) | Patient Health Questionnaire (PHQ-9) | Baseline  Biweekly |
| Anxiety Symptoms (Self-Report) | Generalized Anxiety Disorders Scale (GAD-7) |  |
| Trauma-Related Symptoms  (Self-Report) | Posttraumatic Stress Disorder Checklist for DSM-5 (PCL-5) | Baseline  Treatment End  3-Month Follow-Up  6-Month Follow-Up (Study End) |
| Sleep | Insomnia Severity Index (ISI) |  |
| Wellbeing | World Health Organization-Five Well-Being Index (WHO-5) |  |
| Usability/Feasibility | System Usability Scale (SUS) | 6-Month Follow-Up (Study End) |
| Wearable Data Collection (Passive) | | |
| Physiological, Activity and Sleep Data Collection (Continuous) | Sleep metrics  Cardiovascular metrics  Miscellaneous: Respiratory Rate, Temperature, Stress and Recovery data | Continuous |

**Abbreviations**: GAD-7, Generalized Anxiety Disorder-7; HR, heart rate; HRV: heart rate variability; ISI, Insomnia Severity Index; MADRS, Montgomery-Åsberg Depression Rating Scale; PCL-5, The Posttraumatic Stress Disorder Checklist for DSM-5; PHQ-9, Patient Health Questionnaire-9; SUS, System Usability Scale; WHO-5, World Health Organization-Five Well-Being Index. Details of wearable metrics are provided in Table 3.
