# Supplementary material for: Wearable-Based Assessment for Relapse Prediction Following Repetitive Transcranial Magnetic Stimulation for Depression: Protocol for a Feasibility Study (WARN-D Study)
Source: JMIR Res Protoc. 2026 Sep 21;15:e89466. doi: 10.2196/89466 (PMC13592458; doi:10.2196/89466)
Supplement: Supplementary file 2 [file resprot-v15-e89466-s002.docx]

Multimedia Appendix 2. Schedule of Study Visits

|  | **Study Visits** | | | | | | | | | | | | | | | |
| --- | --- | --- | --- | --- | --- | --- | --- | --- | --- | --- | --- | --- | --- | --- | --- | --- |
| **PROCEDURE or FORM** | **Screening and Baseline**  **(Week**  **-2 or -1)** | **Treatment Phase**  **Weeks 1-6** | | | **Follow-Up Phase** | | | | | | | | | | | |
|  |  | **Week 2** | **Week 4** | **Week 6** | **Month 1** | | **Month 2** | | **Month 3** | | **Month 4** | | **Month 5** | | **Month 6** | |
|  |  |  |  |  | **Wk**  **2** | **Wk4** | **Wk**  **2** | **Wk4** | **Wk**  **2** | **Wk4** | **Wk**  **2** | **Wk4** | **Wk2** | **Wk4** | **Wk2** | **Wk4** |
| ***Visit Duration*** | 3h | 10  min | 10  min | 90  min | 10  min | 10  min | 10  min | 10  min | 10  min | 1h | 10  min | 10  min | 10  min | 10  min | 10  min | 2h |
| Informed Consent | X |  |  |  |  |  |  |  |  |  |  |  |  |  |  |  |
| Demographics | X |  |  |  |  |  |  |  |  |  |  |  |  |  |  |  |
| Structured Clinical Interview for DSM-5 Disorders: Research Version (SCID-5: RV) | X |  |  |  |  |  |  |  |  |  |  |  |  |  |  |  |
| Antidepressant Treatment History Form (ATHF) | X |  |  |  |  |  |  |  |  |  |  |  |  |  |  |  |
| Medical, Psychiatric, and Substance Use History | X |  |  |  |  |  |  |  |  |  |  |  |  |  |  |  |
| Concomitant Medication Record (CONMED) | X |  |  | X |  |  |  |  |  | X |  |  |  |  |  | X |
| Wearable (Oura ring) Set-Up | X |  |  |  |  |  |  |  |  |  |  |  |  |  |  |  |
| *Assessments and Scales - Clinician-Administered* | | | | | | | | | | | | | | | | |
| Montgomery Åsberg Depression Rating Scale (MADRS) | X |  |  | X |  |  |  |  |  | X |  |  |  |  |  | X |
| *Self-Reported Assessments* | |  | | | | | | | | | | | | | | |
| Patient Health Questionnaire (PHQ-9) | X | X | X | X | X | X | X | X | X | X | X | X | X | X | X | X |
| Generalized Anxiety Disorder 7-item scale (GAD-7) | X | X | X | X | X | X | X | X | X | X | X | X | X | X | X | X |
| Insomnia Severity Index (ISI) | X |  |  | X |  |  |  |  |  | X |  |  |  |  |  | X |
| The Post-traumatic Stress Disorder Checklist for DSM-5 (PCL-5) | X |  |  | X |  |  |  |  |  | X |  |  |  |  |  | X |
| World Health Organization-Five Well-Being Index (WHO-5) | X |  |  | X |  |  |  |  |  | X |  |  |  |  |  | X |
| System Usability Scale (SUS) |  |  |  |  |  |  |  |  |  | X |  |  |  |  |  | X |
