# Supplementary material for: Wearable-Based Assessment for Relapse Prediction Following Repetitive Transcranial Magnetic Stimulation for Depression: Protocol for a Feasibility Study (WARN-D Study)
Source: JMIR Res Protoc. 2026 Sep 21;15:e89466. doi: 10.2196/89466 (PMC13592458; doi:10.2196/89466)
Supplement: Supplementary file 3 [file resprot-v15-e89466-s003.docx]

Multimedia Appendix 3. Description of the metrics collected by the study wearable (Oura ring)

| **Domain** | **Metric** | **Frequency or Measurement Unit** |
| --- | --- | --- |
| **Activity** | Activity Score – sum | Daily |
|  | Steps | Daily |
|  | Calories | Daily |
|  | METs | Every 5 minutes |
|  | Activity level | Every 5 minutes |
|  | Workout | Varies (distance in meters, calories, intensity level, heart rate in bpm) |
| **Sleep** | Bedtime latency | Seconds |
|  | Bedtime Start, Bedtime End | Timestamp |
|  | Daily Sleep Score:   - Based on Deep Sleep, Efficiency, Latency, REM Sleep, Restfulness, Timing and Total Sleep | Sleep Score from 0-100 |
|  | Sleep Durations:   - Total time in bed - Total sleep (REM + Deep + Light) - REM Total - Deep Total - Light Total - Awake Total | Seconds |
|  | Sleep Efficiency | Score |
|  | Sleep Hypnogram:   - Integer representation of sleep stages: light, deep, REM, awake | 30-second resolution |
|  | Optimal Sleep Time   - Recommended actions: improve efficiency, earlier bedtime etc. | Timestamp |
|  | Number of Get Ups During Sleep | Count |
| **Cardiovascular** | Cardiovascular age (CVA) (in years) | Age measured in years  Frequency: daily |
|  | Daytime Heart Rate (HR) | Measured for 1 min every 5-10 min |
|  | Heart Rate Average | 1-min resolution |
|  | Heart Rate Lowest | 5-min window |
|  | Heart Rate Time Series | 5-min resolution |
|  | Heart Rate Variability (HRV) Average | Millisecond |
|  | SpO_2_ average | Percentage |
|  | VO_2_ max value | Integer |
| **Miscellaneous** | Respiratory Rate Average | RR (respiratory excursions per minute) |
|  | Temperature:   - Body temperature - Temperature deviation from baseline | Celsius |
|  | Stress and Recovery:   - Daily summary time spent in each level – stressed, engaged, relaxed, restored | Unit: minutes  Frequency: daily |

Abbreviations: MET, Metabolic Equivalent of Task; SpO_2_, peripheral blood oxygenation; VO_2_ max: maximum oxygen uptake.
